# Different Neurophysiological Mechanisms Underlying Word and Rule Extraction from Speech

**DOI:** 10.1371/journal.pone.0001175

**Published:** 2007-11-14

**Authors:** Ruth De Diego Balaguer, Juan Manuel Toro, Antoni Rodriguez-Fornells, Anne-Catherine Bachoud-Lévi

**Affiliations:** 1 INSERM U841 - Equipe 1 Neuropsychologie Interventionnelle, IM3-Paris XII, Créteil, France; 2 Département d'Études Cognitives, École Normale Supérieure, Paris, France; 3 Cognitive Neuroscience Sector, International School for Advanced Studies (SISSA/ISAS), Trieste, Italy; 4 Institució Catalana de Recerca i Estudis Avançats (ICREA) and Facultat de Psicologia, Universitat de Barcelona, Barcelona, Spain; 5 Assistance Publique - Hôpitaux de Paris (AP-HP), Department of Neurosciences, Henri Mondor Hospital, Créteil, France; New York University, United States of America

## Abstract

The initial process of identifying words from spoken language and the detection of more subtle regularities underlying their structure are mandatory processes for language acquisition. Little is known about the cognitive mechanisms that allow us to extract these two types of information and their specific time-course of acquisition following initial contact with a new language. We report time-related electrophysiological changes that occurred while participants learned an artificial language. These changes strongly correlated with the discovery of the structural rules embedded in the words. These changes were clearly different from those related to word learning and occurred during the first minutes of exposition. There is a functional distinction in the nature of the electrophysiological signals during acquisition: an increase in negativity (N400) in the central electrodes is related to word-learning and development of a frontal positivity (P2) is related to rule-learning. In addition, the results of an online implicit and a post-learning test indicate that, once the rules of the language have been acquired, new words following the rule are processed as words of the language. By contrast, new words violating the rule induce syntax-related electrophysiological responses when inserted online in the stream (an early frontal negativity followed by a late posterior positivity) and clear lexical effects when presented in isolation (N400 modulation). The present study provides direct evidence suggesting that the mechanisms to extract words and structural dependencies from continuous speech are functionally segregated. When these mechanisms are engaged, the electrophysiological marker associated with rule-learning appears very quickly, during the earliest phases of exposition to a new language.

## Introduction

Language acquisition requires the identification of the words that compose it and the rules that structure these words. From the initial stages, when speech sounds like an endless stream of nonsense, infants and second language learners are able to segment it into discrete words [1], [2]. In addition, two other important processes have to be carried out: the memorisation of these segmented words and the extraction of the rules embedded in these words. The segmentation step allows for subsequent recognition of words from speech [3], [4] when memory traces of these words have been created. However, the storage of individual words is not sufficient for language acquisition. The form of a word can vary as a function of its dependencies on other elements in an utterance. Thus, learning grammatical/morphosyntactic rules is necessary and permits generalisation to other instances. For example, detecting that the English use of the pronoun “**he**” will add an **–s** at the end of a verb will allow the learner to say “**he** walk**s”**, “**he** stand**s”**, “**he** run**s”**, etc.. Similarly, within words, extracting the co-occurrence of the prefix “un-” with the “-able” ending to create an adjective, allows for the generation of “untreatable”, “unbelievable”, “unbearable”, etc. [5]. Studies in healthy and brain-damaged populations suggest that words and rules are acquired and processed by different neural and cognitive mechanisms [6]–[10]. However, are these two types of information tracked differently since the initial contact with a new language?

Concerning the first step of word extraction from speech, when no semantic or prosodic information is available, it has been suggested that listeners can use a general statistical learning mechanism to segment speech based on adjacent [2] and non-adjacent statistical dependencies between syllables [11]. Morphosyntactic rules are characterised in most languages by dependencies among non-adjacent elements. Thus, if participants are able to use this information to segment, are they able, by the same means, to use this information to extract and generalise the rule carried by those non-adjacent elements? Additional cues, such as the introduction of subtle pauses [11], the presentation of clearly segmented words [12], [13] or the salience of the syllables carrying the critical rule information [14], appear to be necessary to trigger the appropriate mechanisms enabling the extraction of structural information from the speech signal. In addition, the mechanisms for word and rule extraction seem to have a developmental progression. 8-month-old infants can segment words from an artificial language based on the transitional probabilities of syllables forming words [2]. At 7 months of age they are also able to learn and generalise structural information when it includes a repeated syllable in the structure [7]. However, it is not until they are 15 months old that infants start tracking structural dependencies that do not include simple repetitions [12]. This has led some authors to propose that different cognitive mechanisms underlie the ability to extract words and structural dependencies from the speech signal [11]. However, this issue is still controversial [15], [16]. In fact, as previously mentioned, the nature of these mechanisms and their temporal dynamics are still largely unknown.

In the present work, we were interested in studying whether different mechanisms underlie word and rule extraction from speech in the early stages of learning a new language. We used subliminally segmented streams (25 ms pauses between words) in order to study the subsequent processes after segmentation: the creation of memory traces of segmented words and the extraction of structural information from speech. In addition, we wanted to assess the temporal dynamics of the learning process to test whether the two types of information were tracked in parallel or if word learning would precede rule extraction. We approached these two issues by directly tapping the learning process. We combined offline behavioural measures and recordings of electrophysiological responses throughout the learning process and during one online implicit and one offline testing phase. If the underlying mechanisms for the extraction of words and structural information are different, then distinct neurophysiological mechanisms, associated with each type of learning, should be engaged. More precisely, we predicted that, during acquisition, the creation of memory traces for segmented words should induce the appearance of an N400 component, as has been shown in previous experiments [4], [17]. However, a distinct ERP component related to the process of rule-learning should arise in response to the extraction of structural information embedded in the words. As there is no previous ERP work directly tapping the rule-learning process in continuous speech, we did not have a specific prediction for this component. However, if this specific component is related to rule-learning, the group of participants that learn the rule should show an increase in the magnitude of the component through learning. In contrast, no modulation should be present for those participants that do not learn the rule, but have comparable word learning performance. The nature of the evoked components should clarify the cognitive mechanism underlying word and rule extraction. Their temporal development will indicate the time-course of these processes. After acquisition, the presentation of new words violating the rule should elicit lexical (N400) and syntax-related ERP components (possibly an early negativity and a P600). The processing of new words following the rule should be assimilated as possible items in the language, but induce a lexical N400 modulation if they are detected as novel words.

## Methods

### Participants

Twenty-four right-handed volunteers (7 men, mean age 25±6 SD) participated in the study. None of them had a history of neurological or hearing deficits. Written consent was obtained from each volunteer prior to the experiment. The experiment was approved by the local ethics committee of the University of Barcelona. Four participants were discarded from the analysis due to excessive eye-movements.

### Materials and Procedure

**Figure 1:**
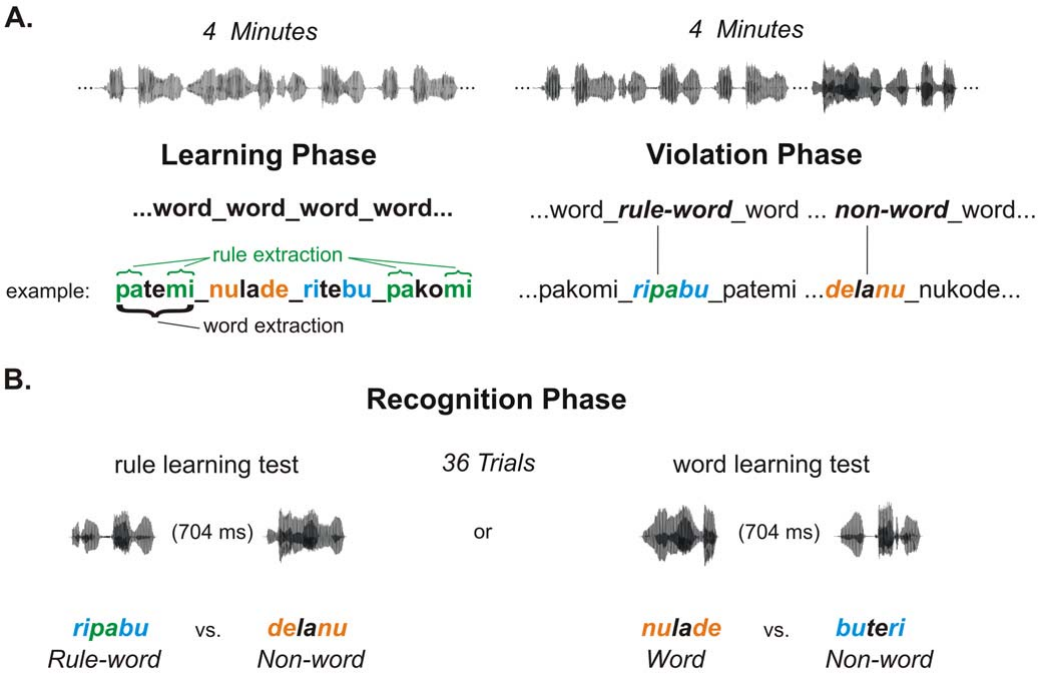
Illustration of the experimental procedure. A. Illustration of the experimental sequence for each language, highlighting the underlying structure of the artificial language. The “_” represents the 25 ms pause between words. After a learning phase lasting four minutes, an online test (*violation phase*) was administered in which new-words, either following the rule or violating it, appeared at random positions in the stream. B. Illustration of the recognition phase. In order to determine whether the participants had learned the words and rules of the language, an offline behavioural test (*recognition phase*) was administered after the violation phase. Half of the streams were tested for *word acquisition*; *rule-learning* was evaluated in the other half using a two alternative forced-choice test. Event-related responses were recorded throughout the whole sequence (*learning, violation and recognition phases*). Each participant was presented with a total of eight languages, thus eight sequences as the one presented here.

Four artificial language streams were created according to the same principle used by Peña et al. [11]. They contained trisyllabic words built following a rule which established that their initial syllable determined their ending (**pa**li**ku**, **pa**se**ku**, **pa**ro**ku**) irrespective of the middle element, thus forming a structure similar to some morphological rules (e.g. **un**believ**able**, **un**treat**able, un**bear**able**) (see Figure 1). There were 3 different frames and the intervening middle syllable could take up to three values, for a total of 9 different words per language (see Table 1). None of the syllables were repeated across languages. Streams and test items were synthesized using the MBROLA speech synthesizer software [18] concatenating diphones at 16 kHz from the Spanish male database (es2) (http://tcts.fpms.ac.be/synthesis/mbrola.html). Words in the language streams had a duration of 696 ms each and were separated by 25 ms pauses, as in Peña et al. [11], to induce the extraction of structural information. They were concatenated in pseudo-random order so that a word was never immediately repeated in the stream. As the same three middle syllables appeared in the three frames of a given language, the transitional probability between the initial and middle syllable, or between this one and the final syllable was 0.33. The transitional probability between the first and the last syllable of every word was 1.0, while the corresponding probability between the last syllable of any word and the first syllable of the following one was 0.5. The material was previously validated in a behavioural pilot experiment to check that words and structural dependencies could be learned from all of the language streams. A filler condition to avoid strategic effects was also created using the same syllables concatenated in random order. In this condition, no words or rules could be extracted. It also included 25 ms pauses every three syllables. In order to have the same length in the different streams and fit the duration to the necessary millisecond precision for the ERP recordings, we used Adobe Audition™ to slightly stretch the audio files.

**Table 1:** Materials used for the different artificial languages.

	Embedded structures	Middle syllables	Word	Non-word	Rule-word
**Language 1**	le__di				
	bo__ma	ka, fi, ro	lerodi	dirole	lemadi
	to__ne				
**Language 2**	ba__gu				
	do__ke	fe, pi, lo	bapigu	gupiba	badogu
	mo__ti				
**Language 3**	pa__mi				
	nu__de	te, la, ko	patemi	mitepa	pabumi
	ri__bu				
**Language 4**	da__lu				
	me__po	na, tu, go	dagolu	lugoda	dabilu
	re__bi				

Middle syllables could be combined with the three structures of the language. Each language had a filler version with a random combination of the same syllables. Word, Non-word and Rule-word columns provide examples of test items.

The experiment involved *learning*, *violation* and *recognition* phases (see Figure 1). Each participant heard a total of four languages and four random streams. The order of presentation was counterbalanced across subjects. A language and its corresponding random version were separated by four intervening streams.

During the *learning phase* of the experiment, each language was presented for 4 minutes leading to 336 word observations per language stream. Participants were told that they would hear a nonsense language and that their task was to pay attention to it because they would be asked to recognize words of this language after listening to it.

The *violation phase* began immediately after a short pause (a few seconds) and lasted four more minutes. This violation phase consisted of the presentation of the same language stream previously heard, but non-words and rule-words were inserted at random positions. Non-words were new items formed with the same three syllables of a previously exposed word in the wrong order: the first and last syllables were placed in the inverse order (see Figure 1). Participants should thus encode the order of presentation of the syllables and their position [19] in order to detect this sequence as an invalid item, as simpler statistical dependencies do not suffice to distinguish them from words. Rule-words were new words with the same initial and final syllable of a word from the exposed language while a syllable corresponding to another word was inserted in the middle position (see Figure 1). Thus, even though these new words followed the structure of words in the artificial languages, the participants had not heard these rule-words before. Each test item (9 rule-words and 9 non-words) appeared twice in the violation phase for each language, leading to 72 rule-word and 72 non-word insertions per participant overall. Thus, violations to the structure of the languages (non-words) represented only 5.3% of the stimuli. There were four to ten intervening words between each test item. In addition, a sample of the electrophysiological responses for words was collected by triggering the presentation of the word appearing 746 ms after the offset of every test item, leading to a sample of 144 observations of the total 1200 words present in this phase. In this implicit test phase, volunteers were not informed about the insertion of test items in the stream during this phase, and they were instructed to continue listening to the speech stream as in the learning phase.

After listening to each stream, participants were behaviourally tested using a two-alternative forced choice test (*recognition phase).* Isolated test items were created and presented in pairs. The two test items of each trial were separated by 704 ms. For half of the streams, participants were tested for *word acquisition,* such that they had to choose between words from the exposed language and non-words in each trial (see Figure 1). For the other half, *rule learning* was evaluated, such that participants had to choose between a non-word and a rule-word. Each test item (9 words, 9 rule-words, 18 non-words) appeared twice, leading to 72 rule-word, 72 word and 144 non-word presentations. Participants were instructed to listen to the two alternative stimuli and wait until an indication on the screen appeared to respond with the right or left button of the mouse.

The experiment was run individually in an electrically and acoustically shielded room on a PC computer using the Presentation Software (http://nbs.neuro-bs.com/). Stimuli were played through Sennheiser (HMD224) headphones connected to the computer, via a Proaudio Spectrum 16 soundcard.

### Electrophysiological data acquisition and analyses

The ERPs were recorded from the scalp throughout the learning, violation and recognition phases using tin electrodes mounted in an electrocap (Electro-Cap International) at 29 standard locations (Fp1/2, Fz, F7/8, F3/4, Fc1/2 Fc5/6, Cz, C3/4, T3/4, Cp1/2, Cp5/6, Pz, P3/4, T5/6, Po1/2, O1/2). Biosignals were re-referenced off-line to the mean of the activity at the two mastoids. Vertical eye-movements were monitored with an electrode at the infraorbital ridge of the right eye. Electrode impedances were kept below 3 kOhm. The electrophysiological signals were filtered with a bandpass of 0.01–50 Hz (half-amplitude cut-offs) and digitalized at a rate of 250 Hz. Trials with base-to-peak electro-oculogram (EOG) amplitude of more than 50 µV, amplifier saturation, or a baseline shift exceeding 200 µV/s were automatically rejected off-line.

Stimulus-locked ERPs were averaged for epochs of 1024 ms starting 100 ms prior to the stimulus. Each analysis was performed for the critical time-windows at parasagittal (PS) (5 levels for the anterior-posterior factor: Fp1/Fp2, F3/F4, C3/C4, P3/P4, O1/O2) and temporal (TE) locations (3 levels for the anterior-posterior factor: F7/F8, T3/T4, T5/T6), including the Hemisphere factor (left, right), in addition to midline (ML) locations (3 levels for the anterior-posterior factor : Fz, Cz and Pz).

Details of the repeated measures analyses of variance are reported in the following section (see Tables 2–4). For all statistical effects involving two or more degrees of freedom in the numerator, the Huynh–Feldt epsilon was used to correct for possible violations of the sphericity assumption. The exact *p*-value after the correction is reported.

Three different analyses were performed on the data corresponding to each phase (*learning*, *violation* and *recognition* phases) of the study. After inspection of the waveforms and in accordance with previous similar studies [4], [17] the following time-windows were chosen:

#### Learning phase

In order to observe learning effects across time, we analysed the learning phase in four 1-minute blocks by averaging all “words” that appeared during every minute of exposition, from their onset, and pooled across the four languages. Two time-windows were chosen for analyses of the learning phase: a 120–220 ms time-window to encompass the P2 component (peaking at 170 ms) and a 350–550 ms time-window for the evaluation of N400 effects.

#### Violation phase

We focused on the 350–550 ms time-window for analysis of the N400 effect. In addition, analyses were performed for critical comparisons according to the specific effects expected in each condition (non-words/rule-word). That is, in the non-word condition, we fixed an early 120–220 ms time-window in order to estimate a possible early negativity effect and a later one at 700–850 ms to assess a late posterior effect.

#### Recognition phase

In the final recognition phase, we were interested in the N400 effects arising in the comparison of the test items and the words in the language stream. Thus, the time-window encompassed the 350–550 ms range.

## Results

### Learning phase

The behavioural measures showed that participants were able to recognise words (62%±13.3; *t*(19) = 4.04, *P*<0.001) and extract the underlying structure from the language streams (54.5%±8.5; *t*(19) = 2.34, *P*<0.03) with better performance in the test for word recognition than for rule learning (*t*(19) = 2.4, *P*<0.02).

The morphology of the ERPs was modulated by the time of exposition. As in previous studies, the creation and consolidation of a memory trace for the segmented words manifested itself in the rapid appearance of an N400 component [4], [17]. The mean voltage values at the 350–550 ms time range were submitted to a repeated measures analysis of variance (ANOVA), including two within subjects factors: Block (1^st^, 2^nd^, 3^rd^, 4^th^ minute) and Anterior-Posterior, and a third within subjects factor Hemisphere (right vs. left) for the PS and TE analyses. The statistical results are summarised in Table 2. These analyses showed a main effect of Block at ML and PS sites. The same analyses applied to the 120–220 time range indicated an increase in the P2 component through the blocks with a main effect of Block. This increase was right lateralized (see Table 2)

**Table 2:** Summary of the statistical results (*F* values) in the ERP learning phase for the N400 (350–550 ms) and the P2 (120–220 ms) component time-windows.

	d.f.		120–220 ms	350–550 ms
Block (1^st^, 2^nd^, 3^rd^, 4^th^)	3,57	ML	3.96+	3.66+
	3,57	PS	4.22++	2.84+
	3,57	TE	3.31+	
Block×Hemisphere	3,57	PS	2.94+	
Block (1^st^, 2^nd^)	1,19	ML		5.2+
Block (1^st^, 3^th^)	1,19	ML	8.62++	
Block×Hemisphere	1,19	PS	6.95+	
	1,19	TE	4.52+	
Block (1^st^, 4^th^)	1,19	ML	4.41+	
	1,19	PS	5.27+	

ML: Midline, TE: Temporal, PS: Parasagittal electrodes; ^+^ *P*<0.05, ^++^ *P*<0.01. Only results with *P*<0.05 are included in the table.

**Figure 2:**
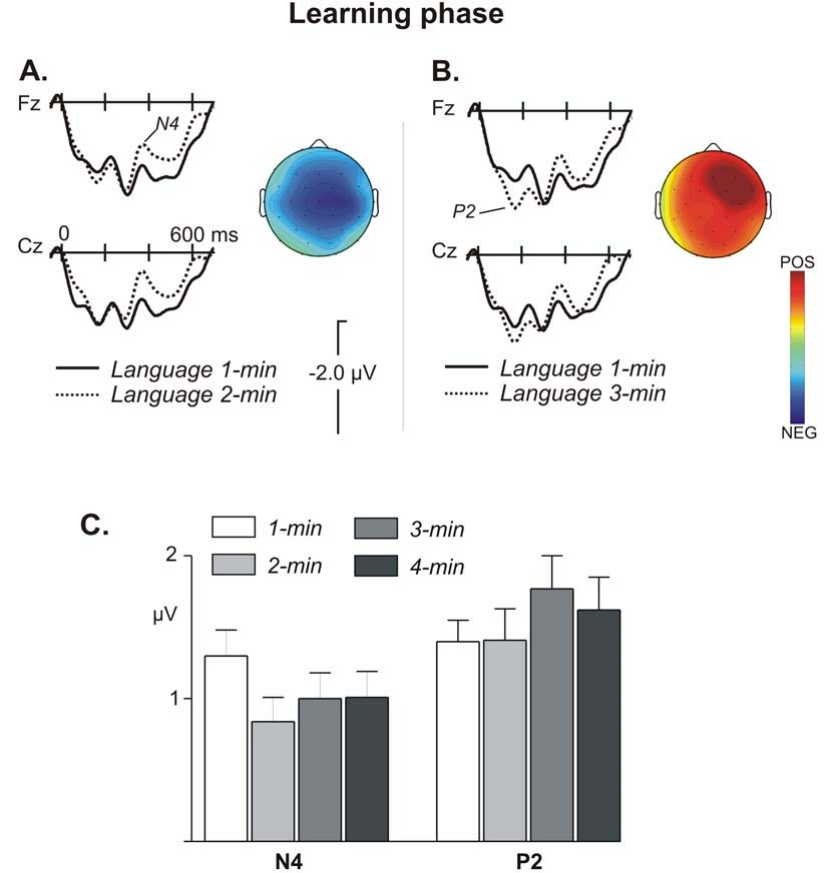
Grand average ERPs at frontal (Fz) and central (Cz) electrode locations for language streams. A. ERP averages comparing the first and second minute of exposition. The ERP signature of the average of words from their onset for the first and second-minute blocks pooled across the four languages is shown. Words in the language streams developed an N400 component during the second minute relative to the first minute. The topography of the difference waveform (subtracting the second from the first minute) showed a central scalp distribution at 50 ms, around the peak of the component (370–420 ms). B. ERP averages comparing the third and first minute. An increase in the amplitude of the P2 component was observed from the third minute. The corresponding difference waveform (third minute minus the first minute) showed a right frontal distribution at 50 ms around the peak of the component (140–190 ms). C. Mean voltage at 50 ms around the peak of the components for the N400 and P2 effects (370–420 and 140–190 ms, respectively) as a function of time at Fz (where both modulations were significant).

A more specific comparison of the blocks showed that the two ERP effects appeared sequentially (Fig. 2A, B, C). The comparison between the first two blocks showed that the N400 was larger in the 2^nd^ minute and the effect was located at central sites (Table 2), being maximal at the right central (C4) location (*F*(1,19) = 10.36, *P*<0.004) (Fig. 2A). None of the interactions with the other factors were significant (all *P*>.1).

The increase in the P2 component appeared later, in the following minutes. This effect was significant at the 3^rd^ minute. A pairwise comparison between the 1^st^ and the 3^rd^ minute showed that the P2 amplitude was larger in the 3^rd^ minute when compared to the 1^st^ minute (Table 2). This effect was larger in the right hemisphere with a maximum at the right fronto-central (Fc2) location (*F*(1,19) = 12.9, *P*<0.002). Further pairwise comparisons showed that the increased P2 amplitude was also significant in the 4^th^ minute (Fig. 2C). The remaining interactions were not significant (all *P*>.1).

**Figure 3:**
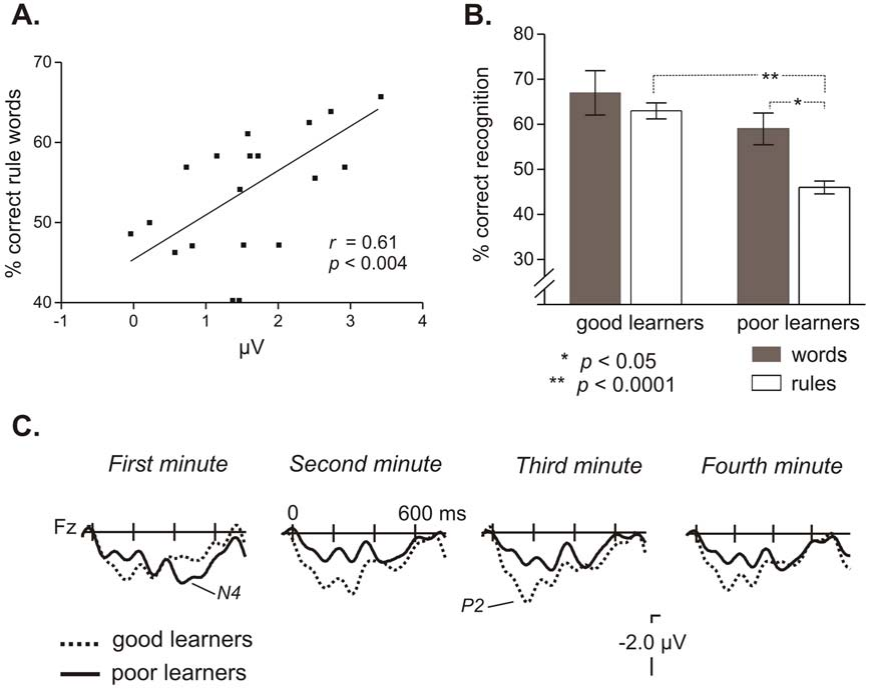
Modulation of the ERP components as a function of rule learning performance. A. Correlation between the mean amplitude of the P2 component at Fz in the third minute of learning (at the 120–220 ms time-window) and the performance on the rule-learning test (N = 20). B. Percentage (± s.e.m.) of correct recognition in the word-learning and rule-learning tests for the groups of good and poor learners (n = 8, in each group). C. ERP averages of the language conditions for each group at a frontal location (Fz), showing the evolution of the differences between groups over the time of exposition (first, second and third minute). While a noticeable increase in the P2 component is shown across time for the good-learners, no modulation is observed for the poor-learners.

The different time-courses and scalp distributions of the P2/N400 effects across the learning phase suggested a possible functional dissociation between the two components. In order to further evaluate this hypothesis, we performed a correlation analysis at a frontal location (Fz; in which both modulations were significant) with the performance during the word-learning and rule-learning tests (recognition phase). While the mean amplitude of the N400 component (350–550 ms) at the 4^th^ minute significantly correlated with the performance in the word-learning test (*r* = −0.51, *P*<0.022), it did not correlate with rule-learning performance (*r = *−0.09). The mean amplitude of the P2 (120–220 ms) in the 3^rd^ minute of exposition strongly correlated with the performance of the participants in the extraction of structural information (Fig. 3A; *r = *0.61, *P*<0.004) while it did not correlate with the word learning test (*r = *0.09). It is also worth mentioning that there was no significant correlation between the performances in the rule-learning and the word-learning tests (*r* = 0.29, *P*<0.22).

In addition, if the P2 component was functionally related to rule-learning, then the group of participants that clearly learned the rule should show an increase in the magnitude of the P2 component through learning. No modulation should be present for those participants that did not learn the rule. Thus, the participants were divided according to their performance in the rule learning test while matched for their word learning performance (Fig. 3B). Planned post hoc comparisons were performed to further disentangle the evolution of the N400/P2 effects as a function of time in each group of learners. The eight participants with the highest performances (>58%) were included in the good-learner group. The eight lowest performers, at chance in the rule-learning test, were assigned to the poor learner group. The remaining four participants with intermediate values were excluded from these analyses. While the mean performance in the rule-learning test was 63%±5 (SD) for the good-learners and 46%±4 for the poor-learners (*t*(14) = −7.84, *P*<0.0001), performance in word learning was comparable in the two groups (good-learners: 67%±14, poor-learners: 59%±10; *t*(14) = −1.39, *P*<0.1).

We performed an analysis of the ERP data introducing the Group factor (good vs. poor learners) and the within-subject factors, Block (1^st^, 2^nd^, 3^rd^, 4^th^ minute) and Anterior-Posterior factors (see Table 3 for the statistical results). For the N400 effect (350–550 ms), significant interactions were encountered between Group×Block and Group×Anterior-Posterior factors. These interactions indicated that good learners showed a larger N400 component at fronto-central locations than poor learners in the first block (Fig. 3C; see direct group comparison in each block at Table 3). Further pairwise comparisons between the 2^nd^ and the 1^st^ minute confirmed that the amplitude of the N400 did not change across time in the good-learners (*F*<1 for ML, PS and TE). Poor learners showed a larger N400 during the 2^nd^ minute (2^nd^ min. vs. 1^st^: ML: *F*(1,7) = 14.2, *P*<0.007; PS: *F*(1,7) = 14.7, *P*<0.006; TE: *F*(1,7) = 5.74, *P*<0.048) (Fig. 3C).

**Table 3 pone-0001175-t003:** Summary of the ERP statistical results (*F* values) for the comparison between groups of the rule (good and poor learners) in the learning phase at the N400 (350–550 ms) and the P2 (120–220 ms) component time-windows.

		d.f		120–220 ms	350–550 ms
**All blocks (1^st^, 2^nd^, 3^rd^, 4^th ^)**	Group	1,14	ML	5.60+	
		1,14	PS	5.96+	
	Block	3,42	ML	2.96+	
		3,42	PS	2.98+	
		3,42	TE	2.98+	
	Group×Block	3,42	ML		3.32+
	Group×AP	4,56	PS		3.81+
	Group×Block×AP	6,84	TE	3.15+	
	Group×AP×H	4,56	PS	3.27+	
**1^st^ minute:**	Group	1,14	ML		4.63+
		1,14	PS	4.66+	
		1,14	TE	4.87+	
	Group×AP	4,56	PS		3.81+
		2,28	TE		3.61+
**2^nd^ minute:**	Group	1,14	PS	4.64+	
	Group×AP×H	4,56	PS	3.93+	
**3^rd^ minute:**	Group	1,14	ML	7.05+	
	Group×AP	2,28	ML	6.88++	
		4,56	PS	6.12++	
		2,28	TE	8.06++	

ML: Midline; TE: Temporal; PS: Parasagittal electrodes. ^+^ *P*<0.05; ^++^ *P*<0.01. Only results with *P*<0.05 are included in the table. AP: Anterior-Posterior; H : Hemisphere; d.f: degrees of freedom.

In the P2 range, the ANOVA with Group (good vs. poor learners), Block (1^st^, 2^nd^, 3^rd^, 4^th^ minute) and Anterior-Posterior factors showed a significant effect of Group at ML and PS sites (see Table 3), indicating that the magnitude of the P2 component was larger for the good learner group. There was also a significant effect of Block indicating an overall P2 increase as time passed. However, most importantly, the evolution of P2 through time was different in the two groups (see Fig. 3C) , with maximal differences in the third minute (see Table 3-bottom). The differences in the P2 effect between groups had a right frontal distribution.

Interestingly, the Block effect in the P2 range showed a significant progressive linear increase as a function of time only for the group of good-learners (ML: *F*(1,7) = 5.68, *P*<0.049; poor learners: *F* (1,7) = 1.6, *P*<.2). This linear increase in the good learner group was maximal at right fronto-central locations, as reflected by the Block by Anterior-Posterior by Hemisphere interaction at PS sites (good-learners: *F*(1,7) = 14.83, *P*<0.006; poor-learners: *F*<1) (Fig. 3C).

### Violation phase

**Figure 4:**
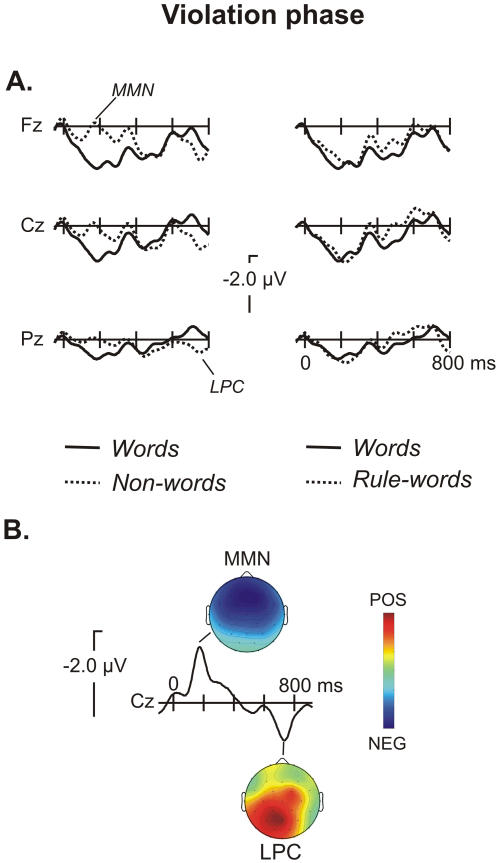
Illustration of the ERP results for the violation phase. A. Left panel: ERP averages from the onset of the presentation of words and new words that violated the previously acquired rule (non-words). An early Mismatch Negativity (MMN) appears, which indicates automatic detection of the rule violation. This negativity is followed by a late positive component (LPC) that could be assimilated into a P600 syntactic component. Right panel: ERP averages from the onset of the presentation of words and new words that violated the previously acquired rule (non-words). B. The difference waveform (subtracting non-words from words) has an MMN effect peaking around 190 ms after the onset of the non-word presentation and a fronto-central distribution. The LPC shows a more left lateralised parieto-occipital distribution that peaks around 720 ms after onset.

Figure 4 shows the ERP signatures from the onset of the trisyllabic word, non-word (violation condition) and rule-word (non-violation condition) and the topographical distribution of the effects centred at the peak. As Figure 4A shows (left panel), the online presentation of new words that violate the rule of the language (*non-words*) elicited a large early negative increase with a fronto-central distribution consistent with a Mismatch Negativity (MMN) effect (Fig. 4B, bottom). The ANOVA of the three conditions (word, non-word, rule-word) showed a main effect of Condition for the 120-220 time-window (ML: *F*(2,38) = 9.57, *P*<0.0005; PS: *F*(2,38) = 10.45, *P*<0.0002; TE: *F*(2,38) = 3.78, P<0.032). Non-words showed a significant larger negativity than words and this effect was frontally distributed (see Table 4 for the summary of statistical results). In order to evaluate the polarity inversion that characterizes the MMN component at the mastoids locations [20], we performed an analysis of variance of condition (Word, non-word) and electrode (non re-referenced left mastoid and right mastoid locations). A main effect of condition was encountered (*F*(1,19) = 4.98, *P*<0.038; mean amplitude words −0.48±0.48 µV; non-words, −0.17±0.56 ) with the expected polarity inversion.

**Table 4:** Summary of the ERP statistical results (*F* values) for the violation phase in the MMN (120–220 ms), the P600 (700–850 ms) components time-windows and for the recognition phase in the N400 (350–800) component time-window.

	d. f.		Violation	Violation	Recognition	Recognition
			120–220 ms	700–850 ms	350–800 ms	350–800 ms
			NW vs. Words	NW vs. Words	NW vs. Words	NW vs. RW
Condition	1,19	ML	14.73++		11.66++	7.56+
	1,19	PS	16.70++		13.14++	9.00++
	1,19	TE	13.97++		8.95++	4.88+
C×H	1,19	PS		5.34+		
C×AP	2,38	ML	13.10++		12.38^+++^	3.67+
	4,76	PS	6.25++		19.83^+++^	
	2,38	TE	6.08+		23.63^+++^	8.22++
C×AP×H	4,76	PS		3.82+	3.79+	13.83++

ML: Midline; TE: Temporal; PS: Parasagittal electrodes. ^+^ *P*<0.05; ^++^ *P*<0.01; *P*<0.001. Only results with *P*<0.05 are included in the table. NW: Non-words; RW: Rule-words; C: Condition; AP: Anterior-Posterior; H: Hemisphere factors; d.f.: degrees of freedom.

After 650 ms, non-words resulted in an increased positivity compared to words (Fig. 4B, left panel). The scalp distribution of this late positive component (LPC) showed a parieto-occipital maximum (Figure 4B, bottom). The ANOVA in the 700–850 ms time-window of the three conditions (word, non-word, rule-word) showed a Condition by Hemisphere interaction at PS locations (see Table 4). This Condition effect was due to a right lateralised larger positivity for non-words than words at posterior sites. The effects at ML sites were not significant.

By contrast, the presentation of new words following the same rule of the language of exposure (rule-words) did not differ from word presentations. There was a slight negative increase at the 350–550 time-window when compared to words, but this difference (Fig. 4A, right panel) was not significant (ML: *F*(1,19) = 2.71, *P*>0.1; PS and TE: *F*<1). Likewise, it was not significant in the more general analysis of the electrodes with greater effects (Cz, C3/4, Cp1/2, Pz, P3/4; *F*(1,19) = 2.68, *P*>.1). The corresponding interactions were also non-significant.

### Recognition phase

**Figure 5:**
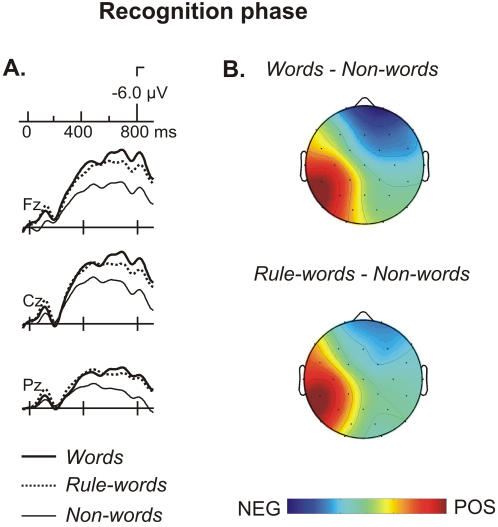
Illustration of the ERP results for the recognition phase A. ERPs averaged from the onset of the presentation of each word in the offline recognition test. While a clear long lasting N400 effect is observed when comparing words and non-words, rule-words did not differ from words. B. Scalp distribution of the N400 effect for non-words compared to words and compared to rule-words. The same topographical distribution of the N400 effect is observed between 350 and 550 ms peaking at fronto-central locations.

Stimulus-locked ERP signatures for the isolated presentation of each test item (words, non-words and rule-words) in the recognition phase are depicted in Figure 5B. A large increase in the N400 component was observed between 350 and 800 ms at fronto-central locations for words and rule-words when compared to non-words. The ANOVA of the three conditions (word, non-word and rule-word) confirmed a main effect of Condition (ML: *F*(2,38) = 6.68, *P*<0.003; PS: *F*(2,38) = 8.26, *P*<0.001; TE: *F*(2,38) = 5.03, *P*<0.012) at the 350–550 time-window. The comparison between word and non-word conditions showed a significant N400 effect (see Table 4 for the summary of statistical results). The effect was greater at frontal electrodes, leading to a Condition by Anterior-Posterior interaction, and was right lateralized at frontal PS sites, peaking at Fp2 (direct pairwise test : *F*(1,19) = 33.59, *P*<0.00001). The same pattern was observed for the comparison between rule-words and non-words with the same right frontal topography (see Table 4). Finally, the differences observed between rule-words and words at this time-window were not significant (ML, PS and TE: *F*<1) (Fig. 5A). Moreover, the interactions with Anterior-Posterior or Hemisphere factors were not significant either (all *P*>.1). The differences were also not significant in a later time window (450–550 ms) that had the greatest differences in amplitude (ML, PS and TE: *F*<1).

## Discussion

By recording electrophysiological responses of individuals learning a new artificial language, we have shown that word learning appears to be functionally different from the extraction of structural relations in the very initial stages of language acquisition. More importantly, our work provides insight to the underlying cognitive mechanisms by showing that specific electrophysiological components are associated with these processes.

### Temporal and functional segregation

It is important to note that, as suggested by Peña et al [11], the introduction of subtle pauses (25 ms) between words probably blocked segmentation by statistical learning, because pauses could be used to perceive words as already segmented tokens. In support of this idea, the N1 segmentation index, which was observed in previous segmentation studies of continuous speech [4], was not observed in our data. Thus, the N400/P200 ERP modulations described in the present study are most probably related to the two processes that have to be carried out once segmentation is overcome: (i) memorisation of the segmented words after repeated presentations and (ii) extraction of the rules embedded in these words.

In our study, exposition to the new language induced two clearly distinct ERP signatures. One signature was a modulation in the N400 component that correlated with the performance in the word-learning test. This modulation was previously reported in speech segmentation tasks that involved learning new nonsense words [4], [17] and in second language acquisition [21], possibly reflecting the construction of a pre-lexical trace for new words. The second signature involves the extraction of the structure that arises from these new words. For the whole group of learners, the P2 modulation correlated with behavioural performance in rule-learning in the third minute of exposition. The fact that this P2 modulation seems to appear at a later stage (third minute) of exposition relative to the N400 suggests that the system needs to “reorganize” the information embedded in the speech signal, chunking it into words, before it can extract the underlying structure.

However, this picture is blurred when the ERP responses of the participants are compared according to their rule-learning performance. A closer look at the group of participants who clearly learned the rules (good learners) shows that from the first minute of exposition, some individuals show an N400, and begin to show a P2 increase. This result suggests that words and rules may be tracked in parallel, by engaging functionally different mechanisms that could be applied to the speech signal simultaneously. Nevertheless, it is worth noting that the evolution of the two components, over the time of exposition to the language, contrast one another. The N400 component shows greater amplitude in the group of good learners relative to the poor learner group during the first minute of exposition, but this difference disappears in the following minutes. Importantly, after this point, the N400 magnitude does not vary through time in either group. By contrast, the P2 component in the good learner group continued to progressively increase, with a maximum in the third minute, correlating with rule-learning performance. Unfortunately, the analysis of the first minute as a single block does not allow us to observe if the increases in the N400 and P2 components developed in parallel, since the beginning of the presentation of the novel speech stream, or whether one mechanism is engaged after the other at earlier stages of the learning process. The different evolution of the two components through time is also interesting because, although there seems to be a functional dissociation between word and rule extraction, the two learning processes seem to be closely related as participants that performed the rule-learning task better had earlier N400 effects.

Aside from the differences in their temporal development, rule-learning, as highlighted by the P2 component, appears to have a different nature from the lexical trace signalled by the N400. The results of the violation and recognition phases point in this direction. A functional differentiation was evident, not only when participants were tested offline, as in previous studies, but also when the test items were inserted in the ongoing-speech and participants were tested implicitly. Importantly, both rule-words and non-words violated the sequence of syllables that characterized the words of the learned language. Thus, both items should have elicited the same ERP response for the presence of the syllable in the unexpected position (at the beginning of the first syllable for non-words and at the beginning of the second for rule-words). However, because the syllable in the unexpected position violated the rule only in the case of non-words, the EEG responses elicited by each were radically different. While non-words elicited an early fronto-central negativity followed by a later posterior positivity, rule-words elicited electrophysiological signatures very similar to those of words from the exposed language.

Friederici and colleagues [22] reported an early negativity with the same distribution observed in the present study. This negativity was followed, as in our study, by a late positivity related to the violations of non-adjacent dependencies, in a study where participants acquired a miniature artificial language. However, in contrast to their work, our participants were not trained or informed about the rule embedded in the language, indicating that these effects also arise in the case of implicit learning procedures. The appearance of an anterior negativity followed by an enhanced positivity (P600) is often reported when combinatorial violations or rule-based morphosyntactic violations in real language are processed (see [23] for a review). This suggests that non-words likely elicited a component associated with syntactic violations [24], [25]. In fact, the topographic distribution of this morphosyntactic negativity effect varies across studies, with left anterior [26], frontocentral [22], [27], bilateral [28], right lateralised [29] and even posterior distributions [30]. These inconsistencies have been attributed to the use of different types of stimuli, different languages, indirect tasks and differences in the individual trials and participants (see [23]).

In the present study, however, we favoured the interpretation that this anterior negativity for non-words is a MMN-like component. Our results show that it is induced by the presentation of a syllable in an unlikely position that violates both word and rule learning. Furthermore, its scalp distribution is consistent with this interpretation and the effect exhibits an inversion of polarity at mastoid locations. This provides further evidence for the elicitation of MMN responses in the case of abstract memorised sequences [31], [32]. Hence, further studies need to elucidate the nature of this type of anterior negativity (as the one observed here and in the Friederici et al. study [22]) when participants are confronted with a new artificial language and whether it reflects the violation of statistical dependencies related to word learning or a more syntactic-like rule violation.

In contrast to non-words, the insertion of rule-words in the speech stream elicited only a slight non-significant negativity compared to words, despite the fact that the middle syllable of these items had never appeared in this position. This might indicate that, once the rule is acquired, listeners maintain the invariant structure of the initial and final syllable and discard the highly variable information (the middle syllable) as irrelevant. The results from the offline recognition phase point in the same direction: while non-words produced a clear long lasting N400 reduction, rule-words appeared to be assimilated as words of the language. Thus, learners detected non-words as impossible items in the learned language, as signalled at the behavioural and neural level, despite the fact that neither rule-words nor non-words appeared in the language previously. Interestingly, these results are very similar to those obtained in first language acquisition [33], suggesting that they may be generalized over the scope of artificial language studies. Finally, it is worth mentioning that the biphasic negative-positive modulations reported earlier were elicited exclusively in online rule-violations (violation phase). When presented in isolation (recognition phase), these violations only induced a lexically-related effect (N400 modulation) comparable to the one found in the acquisition of new words [21], [34]. These differential ERP violation effects in a sentence context compared to those found in isolated words have also been reported in real language processing [35].

### Interpretation of the P2 findings as related to rule-learning

Previous studies have documented the N400 relation to word learning [4], [17], [21], [33], [34], however, the relation found between the P2 modulations and rule-learning is novel. Although the present results do not fully explain the mechanisms that differentiate word and rule learning, we believe that such different ERP signatures may suggest new interpretations and broaden our understanding of them. On the one hand, research from other fields has shown that the P2 component is modulated by perceptual learning and attention [36], [37]. In a recent ERP study, Snyder et al. [38] showed that the amplitude of the P2 auditory evoked-response correlated positively with the perceptual segregation of a single continuous stream of tones in two separate streams. In a similar vein, the P2 appearance was a good correlate of our listeners' perception of initial-final syllable grouping corresponding to rule-extraction. It is interesting to note that a P2 modulation can also be observed in artificial language streams similar to those used in our study, without embedded structural dependencies, only when the words in the stream contain a systematic stress pattern [17].

On the other hand, Peña et al. [11] suggested that the introduction of subtle acoustic cues in the stream, such as small pauses between words, trigger the mechanism responsible for generalization of structural information. Several behavioural studies have detailed further conditions that constrain this type of learning. The different studies include extra information in the speech signal, such as the type of phonetic representations used (i.e., vowels or consonants [39]) or the position of the syllables carrying the rule [14], which help focus attention on the relevant elements that define a given rule. Considering all of this, the P2 modulation as a function of rule-learning might be related to the capture of attention by the cues that facilitate perceptual grouping, when the learners are able to utilize this information properly.

A previous behavioural work suggested that listeners shift their learning strategy from tracking words to employing the underlying structure when the signal contains cues that may facilitate this process [11]. Listeners are able to do this by about 15 months of age [12]. Based on this work, it has been proposed that a shift in the way the speech signal is processed is necessary to extract the rules. Even when using a graded material, such as an increase in the ratio between invariable and variable syllables [13] or in the time of exposition [11], [19], the emergence of the ability to learn the rule is rather sudden, suggesting that listeners shift their learning strategy from a default tendency for word extraction to utilization of non-adjacent dependencies to extract structural information [12]. The P2 modulation may tap this shift. The comparison between our groups of good and poor rule-learners supports this idea. Although the N400 increase appeared in both groups and even though they were able to learn words at the same level of performance, the P2 modulation was only present in participants who learned the rule (see Figure 2A, C). Further research is necessary to tease apart whether both word and rule learning mechanisms are engaged in parallel, such that there is a continuous alternation between the two processes through learning, or if one mechanism is engaged after the other at an early stage of exposition.

Thus, taking previous work and the present results into account, we hypothesize that the P2 increase reflects a perceptual change due to the reallocation of attention to the learning of grouping dependencies between non-adjacent elements. In fact, previous work has shown that the allocation of attentional processing resources is important for the extraction of statistical regularities [40]. These attentional resources may need to be reoriented for rule extraction in order to focus on the common structures observed across words.

If this is the case, maturation of this attentional shifting mechanism might be necessary in order for infants to detect the structural information of speech. This would help to explain the developmental pattern of word extraction. Infants are able to extract words before they attain the ability to exploit structural information [12]. This idea is consistent with later maturation of the brain structures responsible for the control of attention [41]. The existence of this attentional grouping mechanism would not negate the possible existence of similar processes for word and rule extraction, but it points towards the necessary engagement of this additional mechanism during rule-learning.
